# Clinical characteristics of patients with familial idiopathic pulmonary fibrosis (f-IPF)

**DOI:** 10.1186/s12890-019-0895-6

**Published:** 2019-07-18

**Authors:** Ekaterina Krauss, Godja Gehrken, Fotios Drakopanagiotakis, Silke Tello, Ruth C. Dartsch, Olga Maurer, Anita Windhorst, Daniel von der Beck, Matthias Griese, Werner Seeger, Andreas Guenther

**Affiliations:** 1 Universities of Giessen and Marburg Lung Center (UGMLC), Member of the German Center for Lung Research (DZL), Universities of Giessen and Marburg Lung Center (UGMLC), European IPF Registry (eurIPFreg), Klinikstrasse 36, 35392 Giessen, Germany; 2 European IPF Registry & Biobank (eurIPFreg), Giessen, Germany; 3 Cardio-Pulmonary Institute, Giessen, Germany; 4 Agaplesion Lung Clinic Waldhof-Elgershausen, Greifenstein, Germany; 5 Children University Hospital, Campus Hauner, Member of the German Center for Lung Research (DZL), Munich, Germany; 6 Department of Medical Statistics, Justus-Liebig-University of Giessen, Giessen, Germany

**Keywords:** Familial idiopathic pulmonary fibrosis (f-IPF), Idiopathic pulmonary fibrosis (IPF), European IPF registry (eurIPFreg), European IPF biobank (eurIPFbank), Interstitial idiopathic pneumonia (IIP), Diffuse parenchymal lung diseases (DPLD)

## Abstract

**Background:**

The aim of this study was to analyze the relative frequency, clinical characteristics, disease onset and progression in f-IPF vs. sporadic IPF (s-IPF).

**Methods:**

Familial IPF index patients and their family members were recruited into the European IPF registry/biobank (eurIPFreg) at the Universities of Giessen and Marburg (UGMLC). Initially, we employed wide range criteria of f-IPF (e.g. relatives who presumably died of some kind of parenchymal lung disease). After narrowing down the search to occurrence of idiopathic interstitial pneumonia (IIP) in at least one first grade relative, 28 index patients were finally identified, prospectively interviewed and examined. Their family members were phenotyped with establishment of pedigree charts.

**Results:**

Within the 28 IPF families, overall 79 patients with f-IPF were identified. In the same observation period, 286 f-IIP and s-IIP patients were recruited into the eurIPFreg at our UGMLC sites, corresponding to a familial versus s-IPF of 9.8%. The both groups showed no difference in demographics (61 vs. 79% males), smoking history, and exposure to any environmental triggers known to cause lung fibrosis. The f-IPF group differed by an earlier age at the onset of the disease (55.4 vs. 63.2 years; *p* < 0.001). On average, the f-IPF patients presented a significantly milder extent of functional impairment at the time point of inclusion vs. the s-IPF group (FVC 75% pred. vs. FVC 62% pred., *p* = 0.011). In contrast, the decline in FVC was found to be faster in the f-IPF vs. the s-IPF group (4.94% decline in 6 months in f-IPF vs. 2.48% in s-IPF, *p* = 0.12). The average age of death in f-IPF group was 67 years vs. 71.8 years in s-IPF group (*p* = 0.059). The f-IIP group displayed diverse inheritance patterns, mostly autosomal-dominant with variable penetrance. In the f-IPF, the younger generations showed a tendency for earlier manifestation of IPF vs. the older generation (58 vs. 66 years, *p* = 0.013).

**Conclusions:**

The 28 f-IPF index patients presented an earlier onset and more aggressive natural course of the disease. The disease seems to affect consecutive generations at a younger age.

**Trial registration:**

Nr. NCT02951416 http://www.www.clinicaltrials.gov

## Background

Idiopathic Pulmonary Fibrosis (IPF) is a specific form of chronic, progressive fibrosing idiopathic interstitial pneumonia (IIP), a subgroup of diffuse parenchymal lung diseases (DPLD). The disease is characterized by progressive scarring of the lungs, with a consecutive decline in lung function, and is associated with diverse impairments and a poor prognosis [1–3]. Although the pathomechanisms of IPF are not yet fully understood, fibrosis is believed to be caused by chronic epithelial injury especially in genetically susceptible individuals [4]. The known familial occurrence of IPF provides evidence for a genetic background in this disease [5].

The strongest epidemiological evidence for a genetic predisposition is the observation of familial clustering, which has been reported in monozygotic twins raised apart, in consecutive generations of families, and in family members separated at an early age [6]. As opposed to sporadic IPF (s-IPF), familial form of IPF (f-IPF) is defined by the presence of at least two cases of IPF in the same family [7]. In families of IPF patients, 2 to 20% of subjects reported to have a first degree relative with DPLD [7, 8].

In fact, any IIP of known or unknown cases can occur in a familial setting and be related to genetic cause [9]. In general, around 80% of all patients with familial idiopathic interstitial pneumonia (f-IIP) receive the diagnosis IPF, about 10% of f-IIP patients might have the diagnosis of NSIP and another 10% of IIP patients stays as unclassifiable IIP or diagnoses split between remaining IIP entities [10–12].

The reported degree of relation for a diagnosis of f-IPF ranges from 3rd degree, to 2nd degree and to a required first degree relationship [11, 13]. Other authors describe the degree of relationship with “from the same primary family” or “from the same biological family” [14–16]. The relative frequency of f-IPF varies between 0.5 to 9.5% of all IPF patients [9, 17, 18]. An f-IIP appears to be present in 10 to 19.5% of all IIP cases [19, 20].

Many studies have been looking for genetic associations in IPF; during the last years, mutations in components of the shelterin or the telomerase complex as well as in the surfactant proteins A and C have been identified to cause IPF [5, 21]. About a quarter of familial IIP cases can be explained in some studies by specific gene mutations [22].

In general, the telomere length seems to play an important role in IPF development [23, 24]. Naturally, with increasing age, the expression of telomerase in progenitor cells decreases and the chromosomes become steadily shorter [25, 26]. Telomere shortening was found to be below the 10th percentile in 15% of all f-IIP patients and in 10% of s-IIP patients [27]. As a result, cells may be prone to cellular senescence, which in return may provoke fibrogenesis [28]. The presence of short telomere length is associated with poor prognosis in IPF patients [6]. The protein portion of telomerase is encoded by the TERT gene (TERT = telomerase reverse transcriptase), mutations of TERT are the most frequently identified in f-IPF and are present in up to 15% of cases [5, 7, 13]. Other mutations responsible for monogenetic transmission of IPF affect the coding genes for the surfactant proteins C and A2, which result in expression of defective proteins that accumulate in the endoplasmic reticulum (ER) or the lysosomal compartment of alveolar type II cells, thereby causing ER stress and apoptosis [12, 29].

Despite the clear proof of monogenetic transmission in the pathogenesis of f-IPF’s, the penetrance appears to vary substantially, resulting in a clinically apparent disease in some cases within 4 months after birth, whereas others members of the same family are over 60 years of age at first diagnosis. Due to these observations, a “two-hit” concept has been proposed, being based on a genetic predisposition resulting in epithelial injury and second environmental hits, including exposure to known fibrogenic materials (e.g. outdoor pollution and cigarette smoke) [30, 31]. The inheritance pattern of f-IPF has not yet been sufficiently recognized; in general, an autosomal-dominant pathway with reduced penetrance is suspected, although, autosomal recessive inheritance could not be ruled out as well [9, 10, 13].

The intention of this study was to analyse the relative frequency of f-IPF in our patients’ cohort in relation to the sporadic form, as well as to profoundly characterize clinical differences between the f-IPF and s-IPF. We also studied occurrence of the anticipation phenomenon (increasing severity of illness and / or earlier manifestation of the disease in younger generations), as well as the types of inheritance by analysing pedigree charts of our f-IPF cohort.

## Methods

### Collection of phenotyping data

The phenotyping data from IIP patients and their relatives were collected via the European IPF Registry (eurIPFreg) to evaluate the differences between f-IIP and s-IIP groups (with a focus on IPF as a subgroup). All patients were recruited at our UGMLC sites in Giessen and Greifenstein. The purpose was to identify all patients with familial background.

The eurIPFreg is as an Internet-based, multicenter registry interlinked with the European IPF Biobank (eurIPFbank, see also http://www.pulmonary-fibrosis.net) [18]. The data protection concept was approved by local and national networks, such as the TMF (Technology, Methods, and Infrastructure for Networked Medical Research e.V.) and authorities (e.g. Hessian Data Protection Officer, Protocol Nr. 412101 from 25.08.2008). Both, eurIPFreg and eurIPFbank received positive votes from institutional review boards in Europe (e.g. Ethics Committee of Justus-Liebig-University of Giessen; 111/08). Patients were included into the registry starting November 2009 if they were at least 18 years old, had IPF (prevalent or incident cases) or other DPLDs (as comparator group), as diagnosed by the expert site, and had provided written informed consent prior to the inclusion.

The clinical data were collected at the time of enrolment (baseline) and in intervals 3 to 12 month thereafter via patient and physician baseline questionnaires, which can be retrieved upon logging in to our website (http://www.pulmonary-fibrosis.net) [18].

All 28 index patients were diagnosed accordingly ATS/ERS/JRS/ALAT Guidelines 2011 [32]. The definition and evidence level of f-IIP have been established in accordance to the recent publications and guidelines [32–36].

- Grade A - First line relative lives with or died from IPF / NSIP
- Grade B - First line relative lives with or died from DPLD
- Grade C - First line relative died because of a lung disease not further specified

As first line relatives, the biological parents, children and full siblings were taken into account. The f-IPF index patients as well as their families were enrolled into the eurIPFreg. When employing wide range criteria of f-IIP incidence (evidence grade C), 60 index patients were identified. However, after refining the criteria to occurrence of either nonspecific interstitial pneumonia (NSIP) or IPF in one first grade relative (mother, father, sister, brother or child), the search narrowed down to 28 index IPF patients.

The following exclusion criteria were applied: patients under 18 years of age, missing informed consent, death of the patient, other lung diseases apart from IIP, adoption of the patient, as well as evidence level C of f-IPF.

These 28 index patients were prospectively interviewed, examined, and analyzed using the patient and physician questionnaires of the eurIPFreg, including establishment of pedigree charts. The patient questionnaire included patient’s demographics, a detailed medical history and complaints. The physician questionnaire contained data of physical examination and laboratory tests, pulmonary function, HRCT, echocardiography, 6 MWD, co-morbidities as well as other information concerning relevant patient’s diagnosis and therapy [18]. In addition, blood samples were obtained and archived in eurIPFbank.

Adult relatives of the f-IPF index patients were also recruited into the eurIPFreg, and were invited to our DPLD outpatient clinic in order to undergo examination as part of this study. On site, a detailed medical history, a physical examination, a blood collection, a whole body plethysmography, a DLCO test and a blood gas analysis were performed. In total, 52 healthy relatives were included, 26 of whom have carried out all the above-mentioned examinations. One of the 52 family members was diagnosed with an early form of IPF and was re-classified as an affected relative.

### Pedigree charts

The pedigree charts were developed in collaboration with affected families during the clinical presentation of the patient either in our outpatient clinic or via telephone interview. All family members from all known generations were taken into account.

The following data were collected by each individual subject: family relationship, date of birth, date or at least age at death, cause of death, complaints and all known illnesses. In particular, we were looking for the presence of lung diseases. Individuals with known or suspected IIP were a subject to a more extensive survey: the explorative diagnostics with evaluation of the entity and stage of pulmonary fibrosis, age at first manifestation and diagnosis, smoking status, exposure to occupational and environmental risks. In 24 DPLD patients, included in the pedigree charts, only medical history data could be collected. The pedigree charts were generated with the program GenoPro, version 2.5.4.1.

### Comparison of sporadic IIP (s-IIP) to IPF

The data of all s-IIP patients (incl. IPF) recruited at the same sites as the familial form were extracted from the eurIPFreg, applying inclusion and exclusion criteria mentioned above. The following comparison groups were formed: f-IPF vs. s-IPF (*n* = 147), f-IPF vs. s-NSIP (*n* = 17), f-IPF vs. unclassifiable s-IIP (*n* = 69), f-IPF vs. s-COP (*n* = 42).

### Group comparison in lung function tests

The comparison of change over time in forced vital capacity (FVC) was performed between the f-IPF index patients and s-IPF patients. The main requirement for statistical analysis here was the presence of at least five FVC measurements. The required conditions were met by 21 index patients and 54 s-IPF patients. The last recorded FVC value was the last measurement before lost to follow-up, death of the patient, start on antifibrotics, or lung transplantation.

### Statistics

The statistical analyses were carried out in cooperation with the Department of Medical Statistics of the Justus Liebig University Giessen. For the data evaluation, we used software “R Statistics” (R version 3.1.3). The individual groups were independent of each other. The *p*-value for statistical significance was set as *p* ≤ 0.05. For distribution tests, we performed the Shapiro-Wilk test as well as the Anderson-Darling test. For normally distributed variables, an independent two-tailed t-test or Mann-Whitney test were conducted, both with data adjustment. For nominal scaled parameters, the Chi-square test and the Fischer test were performed.

## Results

### Distribution of the diagnoses in f-IPF vs. s-IIP cohort

Of the 28 identified index patients, all had the diagnosis IPF with following levels of diagnostic confidence: 24 definite, three probable, one possible. Together with the 51 affected family members (then total of 79 patients), these patients were diagnosed to have IPF (56%), nonspecific interstitial pneumonia (NSIP, 3%), unclassifiable IIP (u-IIP, 1%) and other DPLD (40%). The IPF diagnosis was done in accordance with an Official ATS/ERS/JRS/ALAT Clinical Practice Guideline 2011 [32]. In those cases in whom we were able to collect the clinical, radiological and pathological information and in whom the IPF diagnosis criteria of the official ATS/ERS/JRS/ALAT Clinical Practice Guideline were met, the diagnosis of IPF could be settled / confirmed. We did not receive the relevant information for all relatives of the index patients. In some cases, especially in case the relative died earlier, we were not able to retrieve reliable further information. In that case a diagnosis of DPLD was considered without any further differentiation. Evidence level A for f-IPF was met by 14 families. The remaining 14 families corresponded to the evidence level B. Compared to them, the s-IIP group (286 patients) showed following distribution of diagnoses: IPF - 51.2%, NSIP - 5.9%, uIIP - 24%, cryptogenic organizing pneumonia (COP) - 14.6%, other DPLD - 4%.

### Characteristics of index patients

In total, 314 IIP patients (both, familial and sporadic forms) were recruited at our two UGMLC sites. The 28 f-IPF index patients represented 8.92% of all IIP cases. The confidence levels of IPF diagnosis were defined accordingly ATS/ERS/JRS/ALAT Guideline 2011 and are shown in Table 1 [32].

**Table 1:** Distribution of confidence levels of IPF diagnosis

Confidence level of IPF diagnosis:	f-IPF index patients (n)	s-IPF patients (n)	all IPF patients (n)
definitive IPF	24	100	124
probable IPF	1	36	37
possible IPF	3	3	6
Information unknown	0	8	8
Total	28	147	161

When relating the diagnosis of all familial cases to the sporadic comparator group, percentage values from 1.4% (u-IIP) up to 23% (IPF) were encountered (see Table 2).

**Table 2:** Prevalence of familial DPLD cases vs. sporadic forms

Diagnosis	Index patients and relatives with IIP (n)	Comparator group (sporadic, n)	Percentage of familial cases in relation to sporadic and familial forms (%)^a^
IPF	44	147	23
NSIP	2	17	10.5
unclassifiable IIP	1	69	1.4
COP	0	42	–
other IIP	0	12	–
All IIP	47	287	14.1
other DPLD^b^	32	–	–
Total	79	287	

Abbreviations: With regard to COP and other IIPs, it was not possible to make a statement about frequency, due to the low prevalence. ^a^ relative to the respective comparator group. Percentages refer to the respective total number (familial and sporadic patients); ^b^ *DPLD* diffuse parenchymal lung disease, *IPF* idiopathic pulmonary fibrosis, *NSIP* non-specific interstitial pneumonia, *COP* cryptogenic organizing pneumonia, *unclassifiable IIP* unclassifiable idiopathic interstitial pneumonia, other IIP = bronchiolitis with associated diffuse parenchymal lung disease, desquamative interstitial pneumonia, acute interstitial pneumonia, lymphoid interstitial pneumonia

All IPF index patients underwent high-resolution computed tomography (HRCT) for diagnosis prior to this study as a part of diagnostic routine. In all cases, the radiological findings were consistent with a UIP, of them definite UIP in 66.7% and possible UIP in 33.3% of the cases. In total, 14 index patients additionally underwent a lung biopsy for histopathological diagnosis; a video-assisted thoracoscopic surgery (VATS) was performed in 11 patients; in all of the 14 cases UIP pattern was seen.

Twenty-one index patients received a bronchoscopy with a bronchoalveolar lavage (BALF); alveolar macrophages were present in 74.8 ± 37.47%, neutrophilic granulocytes in 10 ± 31.81%, eosinophilic granulocytes in 6.54 ± 0.71% and lymphocytes in 8.2 ± 4.95% of the cases (mean values ± SD). In 25 cases autoantibodies (anti-nuclear antibodies = ANA, extractable nuclear antibodies = ENA, rheumatoid factor = RF, CCP, ANCA and basal membrane antibodies) were tested and not found to be abnormal; in general, prior to IPF diagnosis, connective tissue diseases-associated ILD were excluded.

Of the 147 sporadic IPF patients in 97% of the cases function tests were performed, 98% underwent HRCT, 76% performed 6 MWD, 33% of the diagnoses were additionally confirmed by histology. Bronchoscopy was performed in 73% of the patients, echocardiography in 63% of patients.

### Comparison f-IPF and s-IPF groups

Both groups showed no difference in demographics (61 vs. 79% males) and smoking history. The f-IPF group differed by an earlier age at the onset of the disease (55.4 vs. 63.2 years; *p* < 0.001) and broader age range (36–72 years). With an average of 67.0 years, f-IPF index patients died earlier than sporadic cases (71.8 years), although the result was not statistically significant (*p* = 0.059). The results are shown in Table 3.

**Table 3:** Characteristics of f-IPF and s-IPF patients

Patients characteristics	f-IPF index patients *n* = 28	s- IPF *n* = 147	*p*-value
	mean value ± SD	mean value ± SD	
Male [%]	61	79	0.068
Height [cm]	170 ± 8	173 ± 7	0.058
Weight [kg]	80 ± 14	85 ± 16	0.314
BMI [kg/m^2^]	28 ± 5	28 ± 6	0.630
Current tobacco consumption [%]	0	2	1.000
Former tobacco consumption [%]	64	73	0.514
Pack years [n]	14 ± 18	19 ± 20	0.341
Age at onset of symptoms [years]	55.4 ± 10	63.2 ± 11	0.001
Age at first diagnosis [years]	58.3 ± 10	65.1 ± 10	0.001
Age at death [years]	67.0 ± 8	71.8 ± 8	0.059
Death due to IIP [%]	90	95	1.000

Abbreviations: *IIP* idiopathic interstitial pneumonia, *IPF* idiopathic pulmonary fibrosis, *SD* standard deviation, *BMI* body mass index, *pack years* number of packs per day x smoker years, *occupational exposure* occupational contact with potentially lung-damaging substances

The leading onset symptom of f-IPF index patients was dyspnea (79%), similar to that in the s-IPF group. Interestingly, f-IPF patients reported more frequently on persistent, dry cough without expectoration, as compared to s-IPF group (58 vs. 11%, respectively; p < 0.001). During physical examination, “velcro-like” crackles were found in 96% of f-IPF patients, nail widening in 74% and finger clubbing in 13% of all cases. Oxygen supplementation (LTOT) hat 4.76% of the f-IPF patients with mean flow of 2.5 l, as compared to 22.13% of the s-IPF group (mean flow 3.01 l).

On average, f-IPF index patients showed a milder extent of functional impairment at the time point of diagnosis vs. the s-IPF group (VC *p* = 0.027, FVC *p* = 0.011, RV/TLC *p* = 0.026, DLCO *p* = 0.006, pO2 *p* = 0.015). The results are shown in Table 4.

**Table 4:** Results of functional diagnostic in both sporadic and familial IPF cohorts

Lung function tests at initial diagnosis	f-IPF (*n* = 27)	s-IPF (*n* = 143)	*p* value
	mean value ± SD	mean value ± SD	
Spirometry/ Whole body plethysmography			
VC [% predicted value]	74 ± 19	65 ± 18	0.027
FVC [% predicted value]	75 ± 20	62 ± 19	0.011
FEV1/ VC [% predicted value]	104 ± 13	108 ± 16	0.133
TLC [% predicted value]	73 ± 21	69 ± 18	0.293
RV [% predicted value]	87 ± 41	80 ± 30	0.547
RV/ TLC [% predicted value]	97 ± 26	108 ± 25	0.026
Gas exchange			
DLCO/ SB [% predicted value]	55 ± 20	42 ± 18	0.006
Blood gas analysis at rest			
pO2 [mmHg]	75 ± 13	70 ± 10	0.015
O2 Saturation [%]	95 ± 2	94 ± 2	0.259
pCO2 [mmHg]	38 ± 3	39 ± 6	0.262
6 min-walk test			
SpO2 before test [%]	95 ± 2	95 ± 3	0.377
SpO2 after test [%]	96 ± 2	86 ± 8	0.0002
6 MWD [m]	376 ± 140	379 ± 125	0.834
O2 Substitution during the test [l/min]	1 ± 2	4 ± 3	0.003
Right heart echocardiography			
PAPsys [mmHg]	32 ± 13	40 ± 16	0.055

Abbreviations: *f-IPF* familial idiopathic pulmonary fibrosis, *s-IPF* sporadic idiopathic pulmonary fibrosis, *SD* standard deviation, *VC* vital capacity, *FVC* forced vitality capacity, *TLC* total lung capacity, *RV* residual volume, *TLCO/sb* transfer factor in single breath method, *pO*2 partial pressure of oxygen, *pCO*2 partial pressure of carbon dioxide, *SpO*2 oxygen saturation, *VO2max* maximum oxygen absorption, *PAPsys* systolic pulmonal arterial pressure

The most common co-morbidity in the f-IPF group was pulmonary hypertension (26%); obstructive sleep apnea was seen in 7% of the cases, and pulmonary embolism in 7% of the patients. The percentage of patients ultimately treated with antifibrotic drugs was similar in both groups (*p* = 0.208). Lung transplantation was performed in 15% of f-IPF patients, as compared to 5% in s-IPF patients (*p* = 0.094). The average age of death in f-IPF group was 67 years, as compared to 71.8 years in s-IPF group (*p* = 0.059).

The f-IPF group displayed diverse inheritance patterns, mainly autosomal-dominant with variable penetrance. In the f-IPF, the younger generations revealed a tendency for earlier manifestation of IPF vs. the older generation, thus displaying a phenomenon of anticipation (58 vs. 66 years, *p* = 0.013).

### Comparison in FVC decline between f-IPF and s-IPF patients

The decline in FVC was compared between the f-IPF index patients and the s-IPF patients. The statistic requirement for this analysis was the presence of at least five FVC-values, reflecting decline over time. FVC levels measured after therapy start with antifibrotics or after lung transplantation were excluded. These criteria were met by 21 index f-IPF patients and 54 s-IPF patients. The last recorded FVC value was the last measurement before lost to follow-up, death of the patient, start on antifibrotics or lung transplantation.

Patients with f-IPF (*n* = 21) showed an average, relative FVC decline of 0.028% per day (95% CI: 0.018–0.039%). This corresponded to an average, relative decline of 4.94% (95% CI: 3.155–6.71%) in 6 months, or 9.88% per year. The mean percentage starting point of the FVC of f-IPF was 73.63% predicted (95% CI: 65.76–82.47%).

**Fig. 1:**
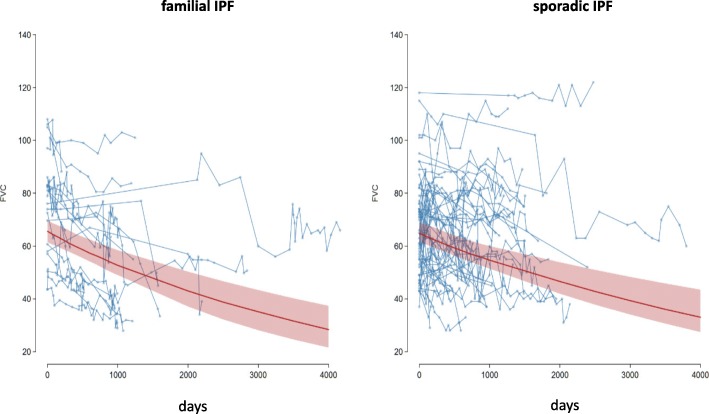
Decline of forced vital capacity (FVC) over time in both groups. Abbreviations: Patients with f-IPF, *n* = 21; s-IPF patients, *n* = 54. Day 0 corresponds to the first lung function test and approximately to the day of the first diagnosis. Each line represents the FVC course of a one patient; the points on the lines mark the individual values of FVC. Red trend line: Interpolated mean percent FVC decline of all patients; red area shows 95% confidence band for model prediction

The s-IPF group (*n* = 54) lost 0.014% (95% CI: 0.009–0.019%) of the FVC on average per day (relative decline). Corresponding to this, the average decline in 6 months was 2.48% (95% CI: 1.568–3.382%), or 4.96% per year. The mean value of the starting point of the FVC in s-IPF group was 62% predicted. However, possibly due to the broad scattering of data, the difference in FVC decline between the groups did not reach statistical significance (*p* = 0.12). In a logarithmic analysis of FVC decline, the starting value in the f-IPF group was 4.30 (95% CI: 4.19–4.41). In contrast, the logarithmic starting point of FVC for sporadic IPF was 4.18 (95% CI: 4.11% - 4.25). The interpolated slope of the FVC for f-IPF was − 0.00028 (95% CI: − 0.00018 – − 0.00039), and for s-IPF group was − 0.00014 (95%-CI: − 0.00009 – − 0.00019). The data are shown in Fig. 1.

### Occurrence of malignancies and evaluation of disease progression in f-IPF group

In general, lung cancer occurred in nine families, in one of the families in two members. Occurrence of multiple malignancies of different organs was found in two families. In six families, patients were rather young when receiving first diagnosis of lung cancer (32, 38, 39, 48, 50 and 50 years, respectively). In one family, it was noticed that all four affected IIP patients died within a very short time after diagnosis due to disease progression. Furthermore, it was observed in five pedigree charts that the younger generation became ill over a decade earlier than the generation of the parents (phenomenon of anticipation).

### Pedigree diagrams of f-IPF patients

Within the families, altogether 79 cases of DPLD including the 28 index patients, as well as 51 further patients with DPLD were detected (44 IPF patients, two NSIP, one unclassifiable IIP, and 32 other DPLD). In fourteen families, two members were affected; another seven families had three affected members per family, six families had four DPLD cases, and one family had six relatives with DPLD.

Pedigree charts could be completed for 25 of the 28 f-IPF patients. In four pedigree charts, a father to son inheritance was observed. In two pedigree charts, the disease appeared to originate from both parental sites. In contrast, in 16 families (ten times inheritance mother to child and six times inheritance father to child), the disease only seemed to originate from one parent. Of these, in one family, children of the same mother (but different father) were affected. Only one generation was affected in seven families (three times exclusively brothers, four times mixed genders).

### Examples of pedigree charts of f-IPF patients

In the following, four pedigree charts are presented as example. The index patient is marked with an arrow. All members suffering from DPLD are marked yellow with a blue border. Patients suffering from another lung disease are marked with a blue border. Family members without DPLD are marked with a magenta border. The relationships in pedigree charts always refer to the index patient.

### Pedigree chart one

**Fig. 2:**
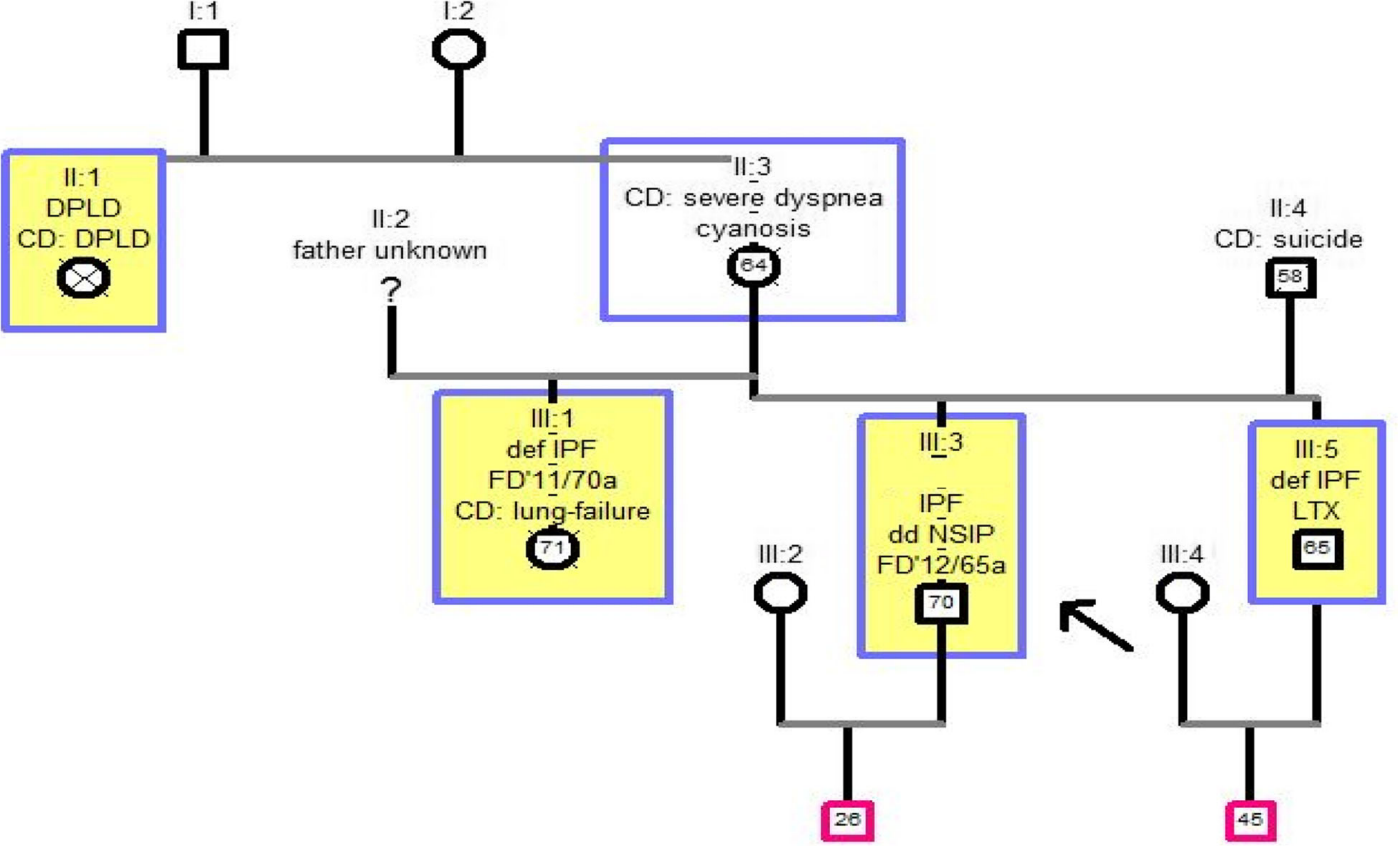
Pedigree chart one. Generations (I-IV) with 13 members, index patient III: 3. Abbreviations: DPLD = diffuse parenchymal lung disease, IPF = idiopathic pulmonary fibrosis, def. = definite, NSIP = nonspecific interstitial pneumonia, CD = cause of death, FD = first diagnosis year / age at first diagnosis in years; LTX = lung transplantation

This family tree comprised four generations with 13 individuals. The grade of evidence of f-IPF was level A, because the index patient (III: 3) had at least one immediate relative diagnosed with IPF or NSIP. In this family, the brother (III: 5) fulfilled this condition. The disease seemed to have its origin on the maternal side. In the mother (II: 3) no diagnosed pulmonary fibrosis could be detected. However, shortly before her death she began suffering from progressive dyspnea and cyanosis without history of nicotine consumption. The aunt of the index patient suffered from pulmonary fibrosis. In the third generation, all three members were affected by an IPF. Inheritance pattern here seems to be autosomal dominant, since all children were affected, despite the fact that they have different biological fathers. The data are shown in Fig. 2.

### Pedigree chart two

**Fig. 3:**
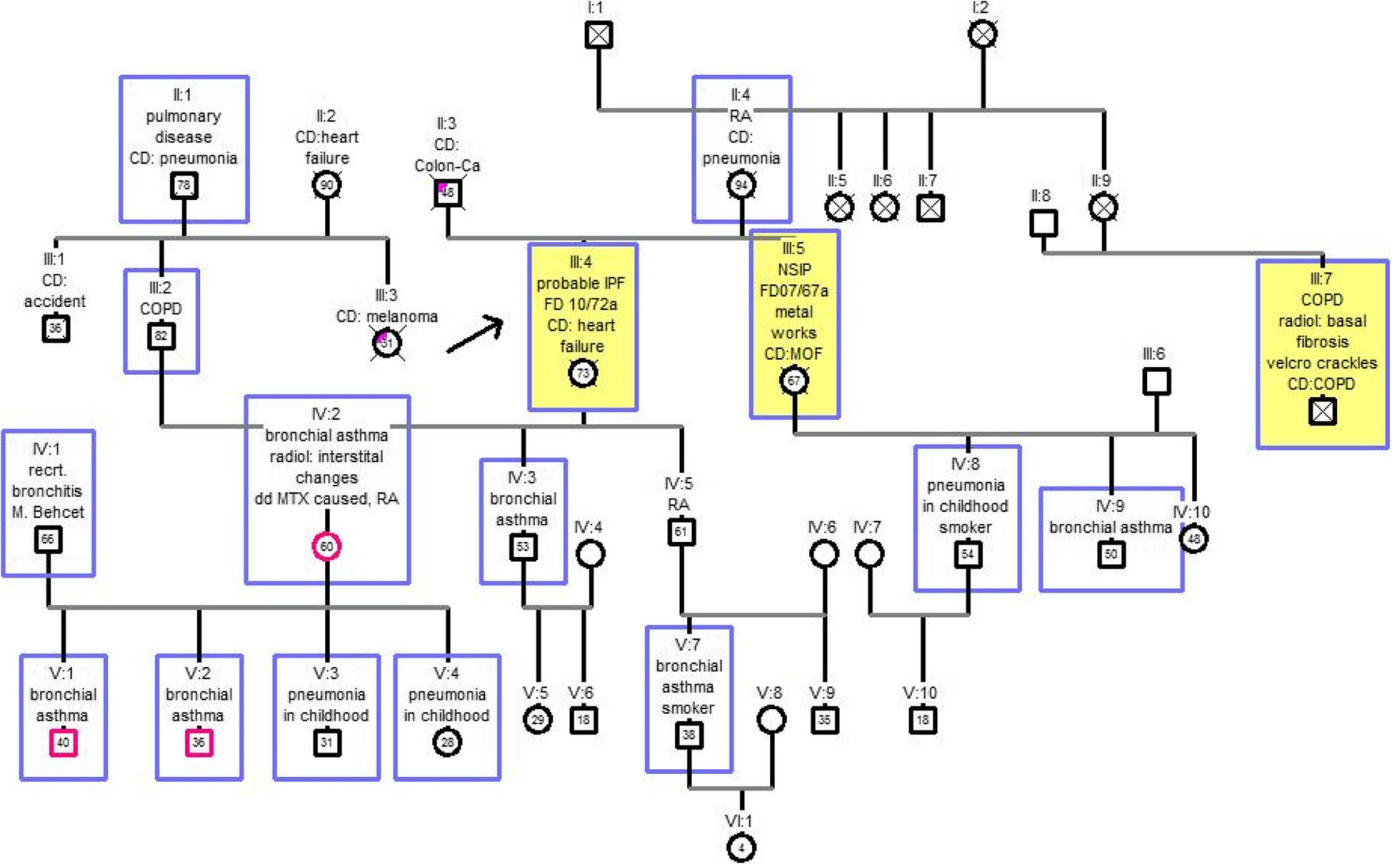
Pedigree chart two. Generations (I-VI) with 39 members, index patient III: 4; IPF = idiopathic pulmonary fibrosis, NSIP = non-specific interstitial pneumonia, CD = cause of death, colon Ca = colon carcinoma, RA = rheumatoid Arthritis, COPD = chronic obstructive pulmonary disease, FD = initial diagnosis year / age at first diagnosis in years, MOF = multi organ failure, radiol. = radiological, recrt = recurrent, DD = differential diagnosis, MTX = methotrexate

This family included six generations with 39 members. The index patient is in position III: 4. There was an evidence level A (index patient III: 4 and sister III: 5). The cousin was also affected by diffuse parenchymal lung disease (III: 7). He was accidentally diagnosed with lung fibrosis because of auscultation of velcro-crackles in the routine checkup. His daughter (IV: 2) also did not have any complaints at the time of first diagnosis of pulmonary fibrosis. In total, ten other blood relatives of the index patient suffered from some form of lung disease. In particular, childhood pneumonia was very common (IV: 8, V: 3, V: 4). Since only one generation was affected with IPF, inheritance mode is challenging to interpret in this example. The results are shown in Fig. 3.

### Pedigree chart three

**Fig. 4:**
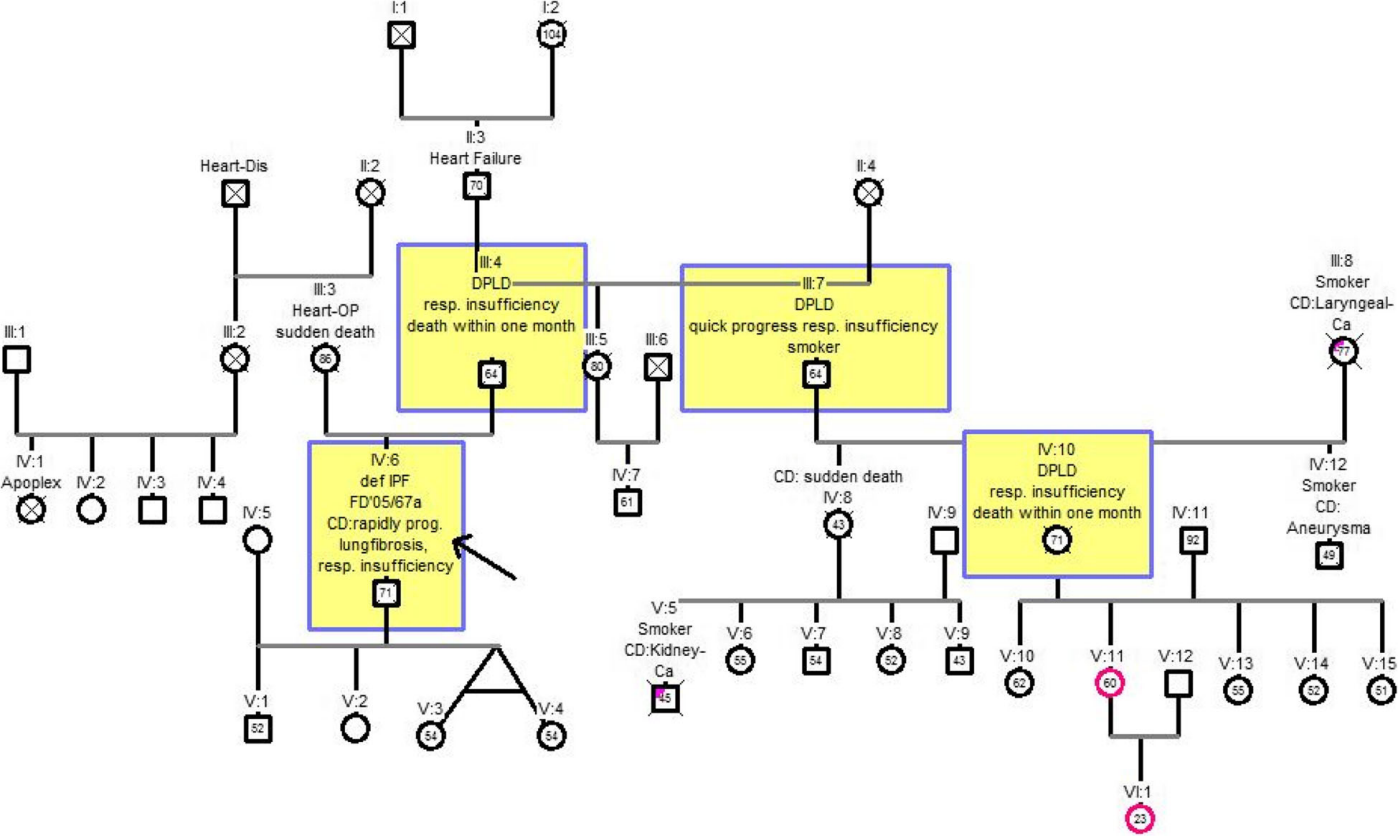
Pedigree chart three. Generations (I-VI) with 42 members, index patient IV: 6. Abbreviations: def. IPF = definitive idiopathic pulmonary fibrosis, Heart-Dis = heart disease; OP = operation; DPLD = diffuse parenchymal lung disease; CD = cause of death; Laryngeal Ca = laryngeal carcinoma; FD = initial diagnosis year / age at first diagnosis in years; rapidly prog. IPF = rapidly progressive idiopathic pulmonary fibrosis; resp. insuff. = respiratory insufficiency

The pedigree chart 3 shown here comprised six generations with 42 family members and fulfilled the evidence level B (IV: six and III: 4). Four members of the family were known to be affected by pulmonary fibrosis (III: 4, III: 7, IV: 6, IV: 10). The index patient (IV: 6) had died of a rapidly progressive lung fibrosis with subsequent respiratory failure at the age of 71 years. More family members had died due to pulmonary fibrosis: his father (III: four at the age of 64), his uncle (III: seven at the age of 64) and a cousin (IV: 10 at the age of 71). The index patient had no siblings. The aunt (III: 5) had died at the age of 80 and had no pulmonary fibrosis during her lifetime. Other children of the DPLD-affected uncle (IV: 8, IV: 12) died early at the age of 43 and 49, respectively, without having developed (or diagnosed) pulmonary fibrosis during their lifetime. The most likely inheritance seems to be an autosomal dominant inheritance, as both father and uncle seem to pass on the gene Fig. 4.

### Pedigree chart four

**Fig. 5:**
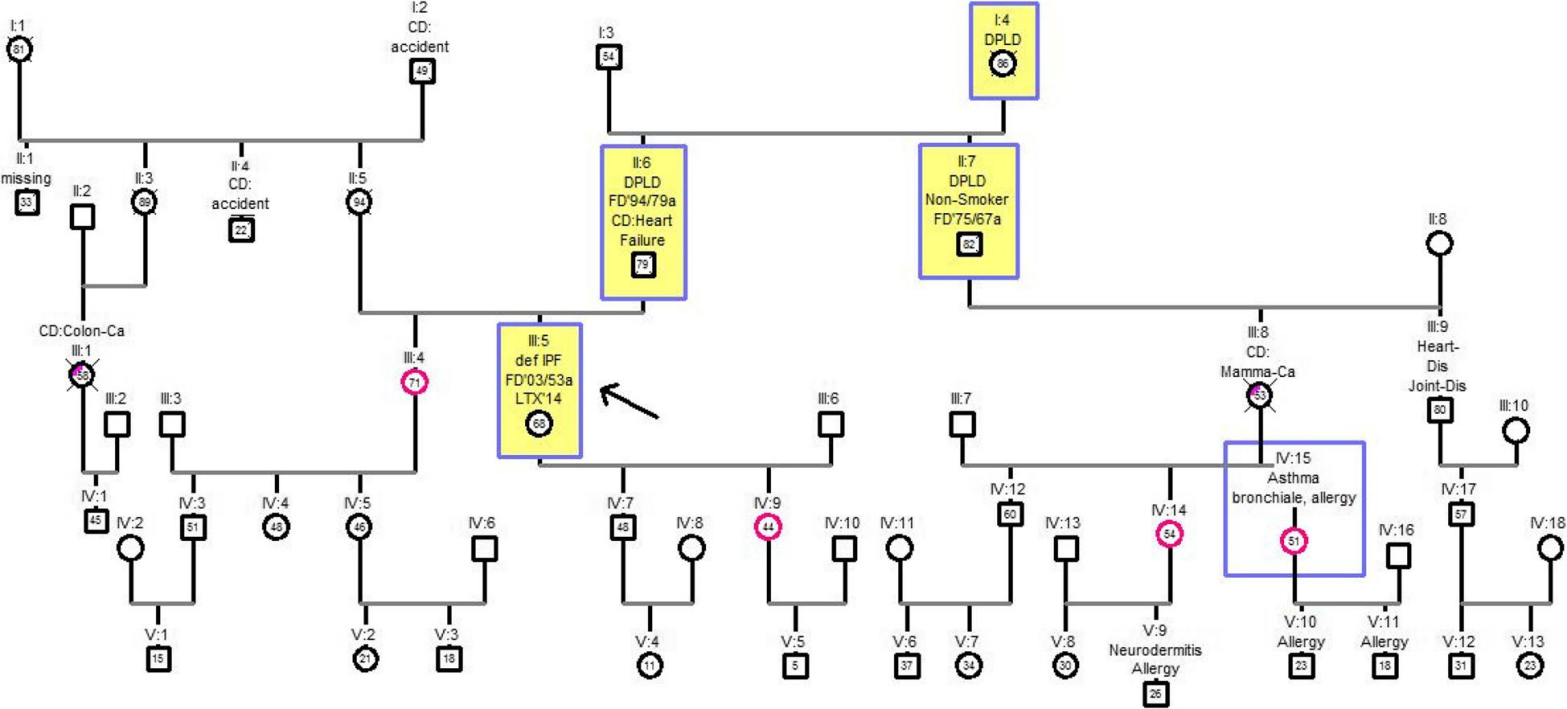
Pedigree chart four. Generations I-V with 53 members, index patient III: 5. Abbreviations: def IPF = definitive idiopathic pulmonary fibrosis; DPLD = diffuse parenchymal lung disease; CD = cause of death; FD = initial diagnosis year / age at first diagnosis in years; colon Ca = colon carcinoma; LTX = lung transplantation; Mamma Ca = breast cancer; Heart Dis = heart disease; Joint Dis = joint disease

The pedigree chart four observed five generations consisting of 53 individuals. Evidence level B was met by the index patient (III: 5) and her father (II: 6). Three generations were affected by pulmonary fibrosis (I, II and III). The sister (III: 4), the daughter (IV: 9) and two nieces (IV 14 and 15) of the index patient had shown no evidence of diffuse parenchymal lung disease at the time of the study. In the oldest generation (I), the grandmother (I: 4) died at the age of 86. In the following generation (II), the father and the uncle died earlier than the grandmother. The father was diagnosed with IPF at the age of 79 years and so the uncle (II: 7) was diagnosed with IPF with 67 years. At age 53, the index patient (III: 5) was diagnosed much earlier than relatives in the previous generations. Due to the rapid progression of the disease, the index patient received a lung transplant at age 64. Up to now, no cases of pulmonary fibrosis have been observed in generations IV and V. This family tree demonstrates the phenomenon of anticipation. The younger the generation, the sooner the disease was diagnosed, but also the sooner the patients died or become lung transplant Fig. 5.

### Generation analysis

The youngest generation was defined as the youngest family member affected by DPLD. Upstream of this generation was the parents’ generation, the grandparents’ generation and so on. The youngest generation in our cohort affected 49 members (40%). In this generation, the age at first diagnosis was 58 ± 11 years (mean ± SD values), which was significantly lower as compared to the year of first diagnosis in the parents’ generation (66 ± 13 years, *p* = 0.013). The affected family members of youngest generation died on average at the age of 66 years. In the parental generation, 25 persons were diagnosed with DPLD (38%), and they died on average at the age of 71 years. The grandparent generation was first diagnosed at the age of 72 and died on average at age 79 (see Table 5).

**Table 5:** Disease manifestation and outcome in different f-IPF generations

	Generations			*p*- value
	Grandparents	Parents	Youngest generation	2./3.Gen.
Affected persons [n]	3	25	49	
Age at first diagnosis [years] [mean value, ± SD]	72 ± -	66 ± 13	58 ± 11	0.013
Age at death (years) [mean value, ± SD]	79 ± 6	71 ± 10	66 ± 9	0.131
Male [%]	33	48	59	0.504

Abbreviations: *SD* standard deviation

## Discussion

In this study, we have provided a detailed report on 28 index f-IPF patients and their families, recruited in the eurIPFreg at our UGMLC centers in Giessen and Greifenstein, as compared to sporadic IPF patients from the same sites. In summary, patients in f-IPF group showed an earlier age at the onset of the disease, reported more frequently on persistent, dry cough without expectoration, and presented with a milder extent of functional impairment at the time point of inclusion, but showed a significantly faster decline of lung function and gas exchange thereafter.

Familial IIP cases in our study were estimated by the presence of at least one direct relative (genetic mother / father, children, full siblings), which suffered or died from IPF / NSIP (evidence level A) or other DPLD (evidence level B). In previous studies, affected family members have often been diagnosed on third-party medical history or questionnaires only [12, 20]. This might be rather vague; as this kind of statement may turn out wrong when assessing the family members’ morbidity in more detail. In our study, five patients reported that a relative was suffering from pulmonary fibrosis, which later proved out wrong.

With regard to f-IPF, the majority of studies indicated that f-IPF does not differ clinically or histologically from its sporadic form. The only difference being observed was an earlier age at onset of familial cases [7, 14, 15]. The mean age at first diagnosis of the s-IPF is known to range at 66–68 years; in case of f-IPF, a significantly lower age (55–62 years) has been described and has been reconfirmed in our study [7, 37].

Loyd at al commented that nine out of 47 (=19%) pulmonary fibrosis patients who underwent lung transplantation at their study center had at least one relative with pulmonary fibrosis [20]. Marshall et al. investigated 21 IPF families with 57 affected members and found out that 0.5–2.2% of IPF cases were familial [38]. Later, Hodgson et al. achieved a percentage of 3.3–3.7% (*n* = 17) for all f-IPF cases [17]. They estimated the prevalence to be even higher, with only about half responding to the questionnaire. In the present study, we found 23% of all IPF and 14% of all IIP patients to have a familial background. Our data therefore seem to fit to the data reported by Loyd et al. and suggest that a familial background is rather common and not a rarity.

By analyzing pedigree charts of f-IPF, we observed mostly autosomal dominant inheritance pathway (16 of 25 pedigrees) with variable penetrance, which has been confirmed by other authors as well [14, 39]. However, a coexistence of different modes or more complex patterns of inheritance, autosomal recessive inheritance, or heterogeneity of hereditary traits cannot be ruled out as well [5, 9, 40].

Bennett et al. retrospectively stratified 46 f-IPF patients according to HRCT pattern and BAL cellular composition and analysed functional and radiological follow-up data of patients with f-IPF, showing that it may manifests with several HRCT patterns with different rates of progression. The study found UIP pattern at HRCT in 54.3% of patients, possible UIP in 21.8% and inconsistent UIP in 23.9% [41]. Our results showed definite UIP pattern in HRCT in 66.7% of patients and possible UIP in 33.3%.

Of note, there were nine families with multiple cases of bronchial carcinoma. This phenomenon has been linked by Wang et al. to a surfactant protein A2 mutation [29] and opens the question if the observed high rate of pulmonary malignancies reported in earlier autopsy studies is largely based on these familial cases or if it rather represents a more common “complication” of IPF in general [42]. An unusual presence of diverse respiratory diseases in four families was also observed.

Within the f-IPF families, the age at onset of disease and of death was found to come down with each generation, hence reflecting the phenomenon of anticipation. The affected family members of youngest generation died on average at the age of 66 years, as compared to 71 years in the parental generation. The grandparent generation was first diagnosed on average at the age of 72 and died on average at age 79, as shown in Table 5. The mortality was linked to the age of diagnosis (*p* = 0.013).

In genetics, anticipation is a phenomenon whereby a genetic disorder is passed on to the next generation, but the symptoms of the disease become apparent at an earlier age with each age group. In some cases, an increase of severity of symptoms was also noted [43]. The proportion of IPF cases between the youngest and the previous generation seems similar in this study (40 vs. 38%). It should be noted that more patients of the younger generation might appear in the future, as they have not yet reached the classic age of onset.

Scholand et al. showed that f-IPF patients have a significantly increased risk of death vs. s-IPF among the first- through third-degree relatives (*p* < 0.001) [44]. The evaluation of the third (grandparents) generation was challenging due to sparse data. The difference in the first year of diagnosis of the second (parents) generation was nevertheless significant (58 vs. 66 years, *p* = 0.013). Some pedigree charts displayed that IPF manifests in the younger generation over a decade earlier vs. parents’ generation. It must be taken into account that the younger generation might be more aware of the disease and could therefore be diagnosed earlier. Nevertheless, the younger generation tends to die earlier (66 vs. 71, *p* = 0.131). Despite the lack of significance, this difference of 5 years on average is considerable, as the younger generation had access to novel therapies, unlike the older generations.

Chibbar at al showed in the analysis of a large family with many IPF patients phenomenon of anticipation in the f-IIP. The oldest generation was diagnosed at the age of 50, with an average of around 39 and the youngest at age 32 [45]. Ravaglia shown a significant difference in onset of IPF between a younger and an older generation in 2014 (58 vs. 74 years) [46]. One possible explanation for this phenomenon could be the increasing telomere shortening over the generations [43]. In addition, the known susceptibility genes lead to premature lung aging because of deteriorated regeneration ability of the lung [39].

The limitations of this research were: the study did not state the proportion of mutated patients, although genetic testing and counseling were proposed to all IPF patients with suspected familial form. The proportion of telomerase-related and surfactant mutations within the cohort of f-IPF is the subject of our upcoming studies. Furthermore, to our understanding, there can only be one index patient per family. These were the 28 patients we had been identifying during their visit at our site. The other fIPF patients were not primary patients of our site. With regard to the concerned family members, we tried our best to get as much information as possible (including CTs) in order to judge on the predominant pattern and the IIP diagnosis. However, this was not always possible. In addition, some of the information which the index patient provided during their visit at our site was not available for their concerned relatives (as they e.g. were already dead). Therefore, we were not able to fully expand the comparison between fIPF and sIPF to all fIPF subjects in the families.

In summary, anticipation of familial IPF can be assumed. In light of the progressive shortening of telomers in successive generations of affected families in those with Terc/Tert mutations, such anticipation phenomenon could be easily explained by the underlying pathomechanisms [47].

Eighteen of the 25 affected relatives in our study had been diagnosed by high-resolution computed tomography (including four patients who also underwent a VATS). Another difference to previous studies is that, in addition to the patients, their healthy relatives were invited for a medical checkup, in order to actively search for an evidence for a diffuse parenchymal lung disease. In particular, young, close relatives and people with mild respiratory symptoms volunteered gladly to take part in this study. This group of family members is precisely the cohort that may have previously unrecognized IPF/IIP; and so the number of false negatives within the families could also be kept to a minimum.

Such early detection may have a significant impact on the life of these patients: prevention of respiratory infections, prevention of exposure to a fumes and gases known to contribute to disease progression (cigarette smoke, outdoor pollution) and early initiation of anti-fibrotic treatment may be mentioned as immediate consequences of such approach and justify a more rigorous screening for concerned family members on clinical grounds.

Whether one needs to conduct genetic screening for routine purpose is a matter of debate and will be subject of position papers in the future: here, the advantage of identifying a disease-causing mutation and the potential to identify family members who even do not show any visible disease at the time of testing contrasts with the burden of knowing that one could develop the disease in a unpredictable time span. We therefore tend to offer clinical screening, with a special emphasis on lung auscultation and screen for velcro rales, to the families of our IPF patients, and not routine genetic testing. For research purpose, however, sequencing should be done and will also be done in our cohort.

## Conclusions

The 28 f-IPF index patients summarized here shown an earlier onset and more aggressive natural course of the disease as compared to s-IPF. The most likely mode of inheritance seems to be autosomal dominant with variable penetrance. In addition, the disease seems affect the consecutive generations at a younger age. Therefore, if f-IPF suspected, a thorough clinical screening of family members should be offered. Further studies will aim to disclose the underlying and causative genetic and epigenetic changes in our cohort of f-IPF patients. This study lays the foundation for evaluating further generations of affected families. The sample size and profound clinical phenotyping and diagnostics of our cohort are the strength of this work.

## Data Availability

The datasets used and analysed during the current study are available from the corresponding author on request.

## Abbreviations

DPLD: diffuse parenchymal lung diseases
eurIPFbank: European IPF Biobank
eurIPFreg: European IPF Registry
f-IPF: Familial idiopathic pulmonary fibrosis
IIP: interstitial idiopathic pneumonia
IPF: idiopathic pulmonary fibrosis
NSIP: nonspecific interstitial pneumonia
UGMLC: Universities of Giessen and Marburg Lung Center
